# Potential immune-modulatory effects of wheat phytase on the performance of a mouse macrophage cell line, Raw 264.7, exposed to long-chain inorganic polyphosphate

**DOI:** 10.5713/ajas.20.0060

**Published:** 2020-05-12

**Authors:** Jeongmin An, Jaiesoon Cho

**Affiliations:** Department of Animal Science and Technology, Konkuk University, Seoul 05029, Korea

**Keywords:** Animal Husbandry, Immunological Effects, Long-chain Inorganic Polyphosphate, Macrophage, Wheat Phytase

## Abstract

**Objective:**

This experiment was conducted to find out the immunological effects of wheat phytase when long-chain inorganic polyphosphate (polyP) treated with wheat phytase was added to a macrophage cell line, Raw 264.7, when compared to intact long-chain polyP.

**Methods:**

Nitric oxide (NO) production of Raw 264.7 cells exposed to P700, a long-chain polyP with an average of 1,150 phosphate residues, treated with or without wheat phytase, was measured by Griess method. Phagocytosis assay of P700 treated with or without phytase in Raw 264.7 cells was investigated using neutral red uptake. The secretion of tumor necrosis factor α (TNF-α) by Raw 264.7 cells with wheat phytase-treated P700 compared to intact P700 was observed by using Mouse TNF-α enzyme-linked immunosorbent assay kit.

**Results:**

P700 treated with wheat phytase effectively increased NO production of Raw 264.7 cells by 172% when compared with intact P700 at 12 h exposure. At 5 mM of P700 concentration, wheat phytase promoted NO production of macrophages most strongly. P700, treated with wheat phytase, stimulated phagocytosis in macrophages at 12 h exposure by about 1.7-fold compared to intact P700. In addition, P700 treated with wheat phytase effectively increased *in vitro* phagocytic activity of Raw 264.7 cells at a concentration above 5 mM when compared to intact P700. P700 dephosphorylated by wheat phytase increased the release of TNF-α from Raw 264.7 cells by 143% over that from intact P700 after 6 h exposure. At the concentration of 50 μM P700, wheat phytase increased the secretion of cytokine, TNF-α, by 124% over that from intact P700.

**Conclusion:**

In animal husbandry, wheat phytase can mitigate the long-chain polyP causing damage by improving the immune capabilities of macrophages in the host. Thus, wheat phytase has potential as an immunological modulator and future feed additive for regulating immune responses caused by inflammation induced by long-chain polyP from bacterial infection.

## INTRODUCTION

Phytase is a type of phosphatase that hydrolyzes phytin [1]. Phytin is a storage compound of phosphate in large quantities in plant seeds and an anti-nutritional factor [1]. The phosphatases are classified as acid or alkaline phytase according to the optimum pH, and depending on the initial position of hydrolysis of the phytate, they are classified as 3-phytase, 6-phytase, or 5-phytase [2]. According to their catalytic mechanisms, phosphatases are classified as histidine-acid phosphatases (HAPs), purple-acid phosphatases, cysteine phosphatases, or β-propeller phytases (BPPhys) [2]. To date, all commercially available phytase belongs to the HAP group, and the fungal-derived phytase, which has been widely used as a feed additive, is affiliated to the HAPs family. In this study, however, wheat phytase is regarded as multiple inositol polyphosphate phosphatase (formerly known as inositol 1,3,4,5-tetrakisphosphate 3-phosphatase), which forms a separate group within the HAP group [2,3]. It has enzymatic substrate hydrolysis properties different from those of fungal phytase [4].

A combination of ten or more inorganic phosphate (Pi) residues by high-energy phosphoanhydride bonds, such as ATP, is called inorganic polyphosphate (polyP), and this linear polymer is found in bacteria, fungi, and some higher eukaryotes [5]. In particular, the long-chain inorganic polyphosphate that is linked to about 200 or more Pi is used in bacterial expression and in the storage of phosphates [6]. It is also required for virulence and survival in many bacteria [7,8]. The gene(*ppk*) that encodes the polyphosphate kinase (PPK), the enzyme responsible for the biosynthesis of polyP from Pi, is important for the long-term survival and virulence of many pathogens, including *Shigella* and *Salmonella* spp. [6,7]. Thus, the inactivation of *ppk* reduces host invasibility and intracellular survival of *Salmonella enterica serovar typhimurium* (*S. typhimurium*) in epithelial cells and macrophages [9]. *S. typhimurium* is a major bacteria of gastrointestinal inflammation caused by food in humans, in poultry it infiltrates into the intestinal tissue and stays there, but in pigs it acts as causative bacteria of chronic enteritis [9]. Recently, the appearance of *S. typhimurium* DT 104-resistant bacteria, caused by the increase of overused antibiotics, made it more important to prevent livestock diseases [9]. *Campylobacter jejuni* is also a major cause of bacterial diarrhea around the world [10]. According to immunological studies, nearly 90 percent of poultry in the United States suffer from *C. jejuni*. Like *Salmonella*, *C. jejuni* needs polyP to maintain its virulence [10]. Thus, modulating the long-chain polyP metabolic pathway can effectively control those bacterial infections in the animal industry. However, previous research mainly focused on short- and medium-chain polyP as a substrate, so little is known about long-chain polyP as substrates [11].

Wheat phytase, a type of exopolyphosphatase (PPX), can hydrolyze polyP into ortho-inorganic phosphate because of its strong, non-specific dephosphorylation property [4]. Therefore, the dephosphorylation of pathogen-derived polyP by wheat phytase changes the metabolism of polyP in pathogens, which might lead to stimulating immune response in immune cells. Interestingly, some studies have shown that polyP, containing 1,150 phosphate residues (P700), similar in size to that in bacteria, combines with high-mobility group box protein-1, a pro-inflammatory cytokine, to elicit a strong inflammatory response in endotheliocytes [12,13]. P700 is reported to induce inflammatory responses in vascular endothelial cells [14]. Thus, wheat phytase might affect the combination of P700 and cytokine and their immune response. To date, however, the reports on polyP have been mainly about endothelial cells, and there have been no reports on macrophages, a typical immune cell. So the purpose of this study is to explore the characteristics of wheat phytase as an immunological modulator in macrophages in response to long-chain polyP, such as P700 substrate that can be released through infection of bacteria.

## MATERIALS AND METHODS

### Reagents and cell culture

Long-chain polyP, P700 with average 1,150 Pi residues was procured from Kerafast (Boston, MA, USA). Wheat phytase and Griess reagent were purchased from Sigma-Aldrich. Cymax mouse tumor necrosis factor α (TNF-α) enzyme-linked immunosorbent assay (ELISA) kit and Raw 264.7 cell (mouse macrophage cell line) were obtained from Ab Frontier and Korean Cell Line Bank, respectively. These cells were maintained in Dulbecco’s modified eagle medium (Gibco Life Technologies, Waltham, MA, USA) containing 10% fetal bovine serum and 1% penicillin-streptomycin (Gibco Life Technologies, USA), and were cultured in a humidified incubator at 37°C with 5% CO_2_.

### Effect of long-chain inorganic polyphosphate treated with wheat phytase on nitric oxide production of Raw 264.7 cells

At first, we seeded Raw 264.7 cells onto 96-well plates at a concentration of 2×10^4^ cells per well and cultured them until 80% confluency. P700 (500 mM) was incubated at 37°C for 4 h with or without wheat phytase (42.9 mU/mL). After the incubation, we applied aliquots (10 μL) of the reaction mixture (0.1 mL) to the cells at 37°C for 1, 6, 12, and 24 h in the CO_2_ incubator. In addition, we treated aliquots (10 μL) of the reaction mixture containing different concentrations of P700 with or without wheat phytase (42.9 mU/mL) to the cells with each final concentration of 0.1, 1, 2.5, 5, 25, and 50 mM P700 at 37°C for 12 h in the CO_2_ incubator. The levels of nitric oxide (NO) released into the culture media were measured at optical density at 540 nm (OD_540 nm_) using the Griess method [15].

### Effect of long-chain inorganic polyphosphate treated with wheat phytase on phagocytic activity of Raw 264.7 cells

Raw 264.7 cells were initially seeded onto 96-well plates at a concentration of 2×10^4^ cells per well and were cultured until 80% confluency. We did a phagocytic activity assay at 37°C for 4 h in 0.1 mL of a reaction mixture consisting of P700 with or without wheat phytase. We treated aliquots (10 μL) of the reaction mixture containing 500 mM P700 with or without wheat phytase (42.9 mU/mL) to the Raw 264.7 cells at 37°C for 1, 6, 12, and 24 h in the CO_2_ incubator. In addition, we treated aliquots (10 μL) of the reaction mixture made up of different concentrations of P700 with or without wheat phytase (42.9 mU/mL) to the cells with each final concentration of 0.1, 1, 2.5, 5, 25, and 50 mM P700 at 37°C for 6 h in the CO_2_ incubator. Phagocytic activity was measured at OD_540 nm_ using neutral red uptake [16].

### Effect of long-chain inorganic polyphosphate treated with wheat phytase on the release of TNF-α in Raw 264.7 cells

First of all, Raw 264.7 cells were initially seeded onto 96-well plates at a concentration of 2×10^4^ cells per well and were cultured until 80% confluency. P700 (1 mM) was incubated at 37°C for 4 h with or without wheat phytase (42.9 mU/mL), and aliquots (10 μL) of the reaction mixture were added to the cells at 37°C for 0.5, 1, 3, and 6 h in the CO_2_ incubator. In addition, we treated aliquots (10 μL) of the reaction mixture containing different concentrations of P700 with or without wheat phytase (42.9 mU/mL) to the cells with each final concentration of 2, 10, 50, and 100 μM P700 at 37°C for 1 h in the CO_2_ incubator. The levels of TNF-α secreted into the culture media were assayed at OD_450 nm_ using a mouse TNF-α ELISA kit according to the manufacturer’s instructions.

### Statistical analysis

Statistical analysis was done by one-way ANOVA using PROC GLM (SAS 9.4, SAS Institute Inc, Cary, NC, USA) to test for significant differences between treatments with the Duncan’s multiple-range test. The probability levels used for statistical significance were p<0.05. The results are presented as the mean and standard error from three experiments.

## RESULTS

### Effect of long-chain inorganic polyphosphate treated with wheat phytase on nitric oxide production of Raw 264.7 cells

**Figure 1:**
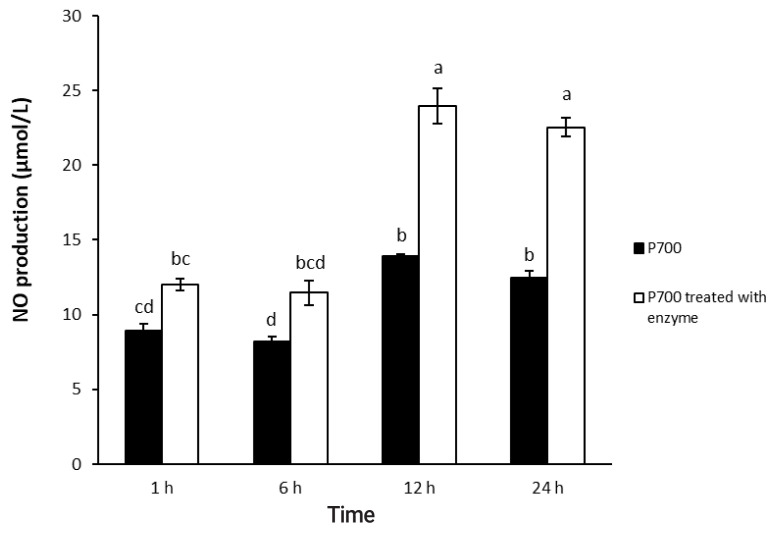
Nitric oxide production by Raw 264.7 macrophage cells exposed to long-chain inorganic polyphosphate treated with wheat phytase during different times. Data are expressed as the mean and standard error from three experiments. ^a–d^ Means lacking common superscripts differ significantly (p<0.05).

**Figure 2:**
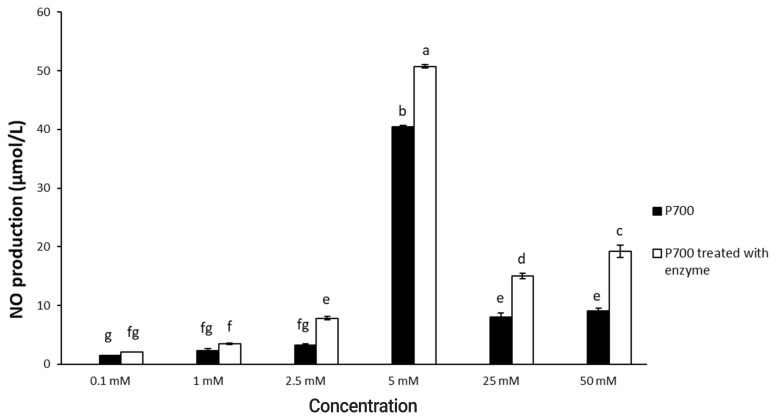
Nitric oxide production by Raw 264.7 macrophage cells exposed to various concentrations of long-chain inorganic polyphosphate treated with wheat phytase. Data are expressed as the mean and standard error from three experiments. ^a–g^ Means lacking common superscripts differ significantly (p<0.05).

As shown in Figure 1, 2, P700 treated with wheat phytase increased NO production of Raw 264.7 cells more than did intact P700. At 12 h, compared with intact P700, P700 treated with wheat phytase promoted NO production of macrophages to approximately 172% (Figure 1). Wheat phytase promoted NO production most strongly at the 5 mM concentration of P700 (Figure 2).

### Effect of long-chain inorganic polyphosphate treated with wheat phytase on phagocytic activity of Raw 264.7 cells

**Figure 3:**
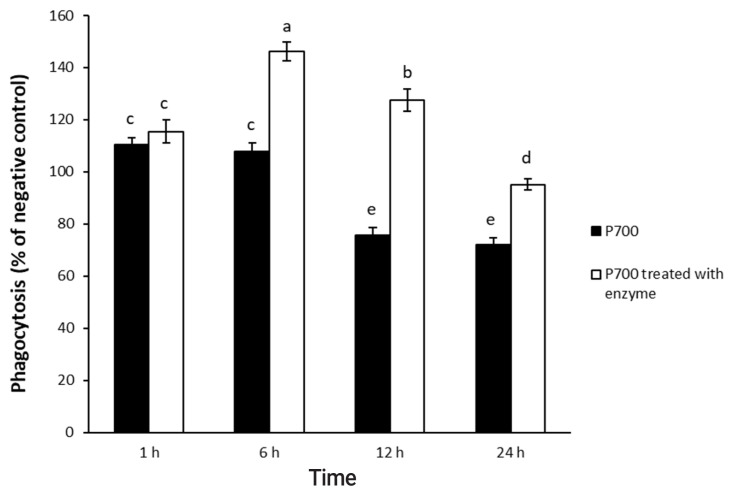
Phagocytic activity by Raw 264.7 macrophage cells exposed to long-chain inorganic polyphosphate treated with wheat phytase during different times. Data are expressed as the mean and standard error from three experiments. ^a–e^ Means lacking common superscripts differ significantly (p<0.05).

**Figure 4:**
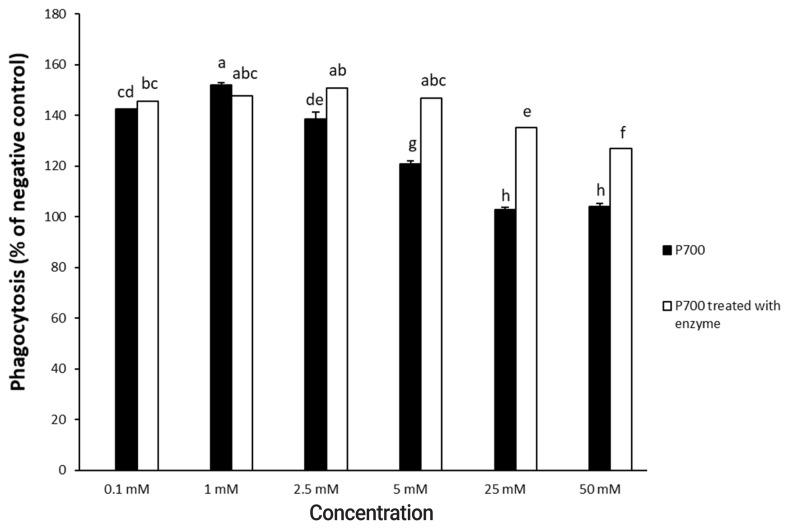
Phagocytic activity by Raw 264.7 macrophage cells exposed to various concentrations of long-chain inorganic polyphosphate treated with wheat phytase. Data are expressed as the mean and standard error from three experiments. ^a–h^ Means lacking common superscripts differ significantly (p<0.05).

P700 treated with wheat phytase stimulated phagocytosis of macrophages. At 6 h, P700 treated with wheat phytase displayed the maximal phagocytic activity of Raw 264.7 cells and at 12 h, wheat phytase promoted phagocytosis of Raw 264.7 cells by about 1.7-fold over that of the intact one (Figure 3). The phagocytosis of all P700 with wheat phytase at concentrations above 5 mM of P700 was increased by more than 1.2-fold over that of the intact P700 (Figure 4).

### Effect of long-chain inorganic polyphosphate treated with wheat phytase on the release of TNF-α in Raw 264.7 cells

**Figure 5:**
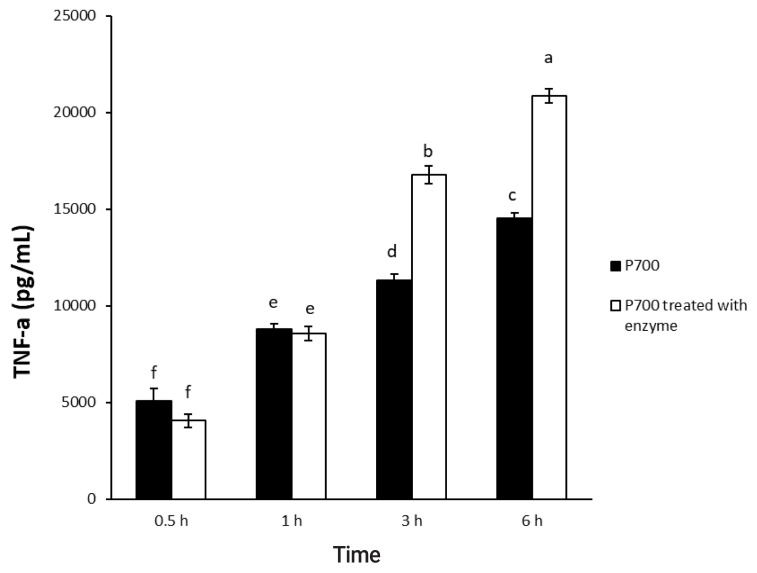
Effect of long-chain inorganic polyphosphate treated with wheat phytase on tumor necrosis factor α (TNF-α) secretion in Raw 264.7 cells during different times. Data are expressed as the mean and standard error from three experiments. ^a–f^ Means lacking common superscripts differ significantly (p<0.05).

**Figure 6:**
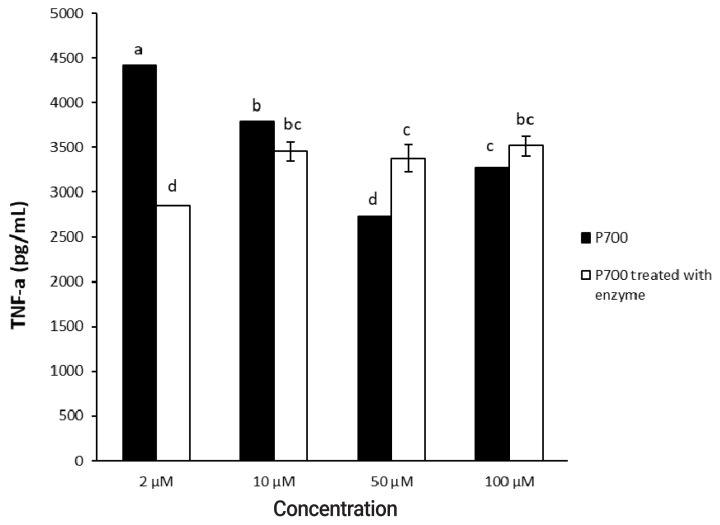
Effect of various concentrations of long-chain inorganic polyphosphate treated with wheat phytase on tumor necrosis factor α (TNF-α) secretion in Raw 264.7 cells. Data are expressed as the mean and standard error from three experiments. ^a–d^ Means lacking common superscripts differ significantly (p<0.05).

In a time-dependent manner, both intact P700 and P700 treated with wheat phytase increased TNF-α secretion from macrophage cell line of Raw 264.7 cells (Figure 5). In particular, the TNF-α release was increased by about 143% over that from intact P700 after 6 h of the exposure of P700 treated with wheat phytase. In addition, at the concentration of 50 μM P700, the wheat phytase increased the secretion of cytokine, TNF-α, by 124% over that of the intact P700 (Figure 6).

## DISCUSSION

In animal husbandry, long-chain polyP-mediated infection by pathogenic bacteria can be controlled by application of immune-modulatory supplements like probiotics, herbal and essential oil [17,18]. But they have only indirect effects on the targets and stimulate other immune cells in the body that may be obscurable [17]. The potential of wheat phytase as an effective immunological modulator is reported in terms of various immunological response assays in this study.

Nitric oxide, an important molecule that removes tumor cells and pathogenic microorganisms, regulates the activation of neutrophils, mast cells, and natural killer cells involved in inflammatory responses [19]. In this study, P700 with added wheat phytase stimulated more NO release than did intact P700. A typical immune activity of macrophages is phagocytosis, the main process that removes pathogenic microorganisms and wastes from the blood or tissue fluid at phagocytes, such as macrophages or polymorphonuclear leukocyte [20]. Phytase-treated P700 increased the phagocytosis in macrophages by dephosphorylating polyP to inorganic phosphate. In recent years, several studies on the interaction between macrophages and phosphate have suggested that phosphates, such as tris-(1,3-dichloro-2-propyl)-phosphate and basic calcium phosphate, increase the intracellular reactive oxygen species level in macrophages and promote macrophage polarization, respectively [21,22]. In addition, sphingosine 1-phosphate (S1P)/S1P receptors (S1PRs) promoted polarization of BMMs (monocyte/macrophages derived from mouse bone marrow) [23]. Presumably, the release of inorganic phosphate from P700 by wheat phytase might stimulate the production of NO and phagocytosis in macrophages. Macrophages secrete various inflammatory cytokines, a type of extracellular signaling molecule that regulates overall innate and adaptive immunity actions, such as infection, inflammation, and B and T cell differentiation and proliferation that are caused by pathogenic microorganisms or viruses [24]. Among these cytokines, pro-inflammatory cytokine TNF-α promotes immune response early in the immune response [25]. The TNF-α secretion of macrophages is modulated by probiotics like *lactobacillus* [26]. The secretion of this cytokine in macrophages is also stimulated by some kinds of phosphatase. Previous studies have shown that protein tyrosine phosphatase-1B regulates the inflammatory cytokines, such as interleukin-1β (IL-1β), IL-6, and TNF-α in Raw 264.7 cells [27]. Furthermore, protein tyrosine phosphatase receptor type O, which belongs to the phosphor tyrosine phosphatases family, promoted the inflammatory response in macrophages [28]. Like these phosphatases, P700 treated with wheat phytase increased the immune response of macrophages induced by TNF-α. Therefore, in this study, wheat phytase facilitated the production of pro-inflammatory cytokine and might stimulate the immune response of macrophages.

In conclusion, wheat phytase could be an immunological modulator in the feed industry, because it increased the NO production, phagocytosis, and cytokine secretion of macrophages. Thus, it can enable macrophages to improve the health and welfare of the host livestock. There are some studies showing that phytase increases the number of lymphocytes and antibodies of a broiler, so attention is needed for its role as an immunological regulator beyond its typical role as a digestive assistant [29]. However, there are various factors that can influence the activity of phytase, such as the optimal pH range, phytase resistance, animal species, and age [1]. The information available on the catalytic properties of polyP hydrolase is very limited. Therefore, this study of wheat phytase as a new regulator of polyP-mediated inflammatory response is very important considering the academic and industrial implications and the ripple effects of the results of the research.
